# Detection fidelity of AR mutations in plasma derived cell-free DNA

**DOI:** 10.18632/oncotarget.14926

**Published:** 2017-01-31

**Authors:** Alexa Goldstein, Patricia Valda Toro, Justin Lee, John L. Silberstein, Mary Nakazawa, Ian Waters, Karen Cravero, David Chu, Rory L. Cochran, Minsoo Kim, Daniel Shinn, Samantha Torquato, Robert M. Hughes, Aparna Pallavajjala, Michael A. Carducci, Channing J. Paller, Samuel R. Denmeade, Bruce Kressel, Bruce J. Trock, Mario A. Eisenberger, Emmanuel S. Antonarakis, Ben H. Park, Paula J. Hurley

**Affiliations:** ^1^ The James Buchanan Brady Urological Institute, Department of Urology, Johns Hopkins School of Medicine, Baltimore, MD, USA; ^2^ The Department of Oncology, Johns Hopkins School of Medicine, Baltimore, MD, USA; ^3^ The Sidney Kimmel Cancer Center, Johns Hopkins School of Medicine, Baltimore, MD, USA; ^4^ The Department of Pathology, Johns Hopkins School of Medicine, Baltimore, MD, USA; ^5^ The Whiting School of Engineering, Department of Chemical and Biomolecular Engineering, Johns Hopkins University, Baltimore, MD, USA

**Keywords:** next generation sequencing, droplet digital PCR, circulating tumor DNA, androgen receptor, DNA polymerase

## Abstract

Somatic genetic alterations including copy number and point mutations in the androgen receptor (*AR)* are associated with resistance to therapies targeting the androgen/AR axis in patients with metastatic castration resistant prostate cancer (mCRPC). Due to limitations associated with biopsying metastatic lesions, plasma derived cell-free DNA (cfDNA) is increasingly being used as substrate for genetic testing. *AR* mutations detected by deep next generation sequencing (NGS) of cfDNA from patients with mCRPC have been reported at allelic fractions ranging from over 25% to below 1%. The lower bound threshold for accurate mutation detection by deep sequencing of cfDNA has not been comprehensively determined and may have locus specific variability. Herein, we used NGS for *AR* mutation discovery in plasma-derived cfDNA from patients with mCRPC and then used droplet digital polymerase chain reaction (ddPCR) for validation. Our findings show the *AR* (tTC>cTC) F877L hotspot was prone to false positive mutations during NGS. The rate of error at *AR* (tTC>cTC) F877L during amplification prior to ddPCR was variable among high fidelity polymerases. These results highlight the importance of validating low-abundant mutations detected by NGS and optimizing and controlling for amplification conditions prior to ddPCR.

## INTRODUCTION

The use of cell-free DNA (cfDNA) as a “liquid biopsy” to detect cancer biomarkers is an emerging and promising area of cancer research. Small amounts of circulating cfDNA can be detected in plasma from healthy individuals [1], is often highly elevated in cancer patients [1] and correlates with disease burden [2–4]. Advancements in technologies such as next generation sequencing (NGS) and droplet digital polymerase chain reaction (ddPCR) have facilitated detection of low-abundance cancer-specific genomic alterations in the blood. Compared to biopsy, cfDNA is minimally invasive, captures a more comprehensive representation of disease heterogeneity, and is more facile for monitoring therapy resistance-associated genetic alterations [5].

Somatic alterations in steroid hormone receptors such as estrogen receptor (ER)α, *ESR1* and androgen receptor, *AR* have been identified following progression on targeted therapies [6–12]. The feasibility of using plasma derived cfDNA to determine receptor hormone status in patients with advanced cancer has been demonstrated recently [13–17]. Analysis of *AR* status in cfDNA in men with metastatic castration resistant prostate cancer (mCRPC) showed that *AR* gene aberrations such as gene amplification or ligand binding domain (LBD) mutations are associated with worse outcomes following next generation therapies that inhibit the androgen/AR axis such as abiraterone and enzalutamide [14, 15, 17]. While validation is still needed, these early studies highlight the clinical potential for using plasma cfDNA to detect *AR* status as a biomarker for therapy resistance.

Translation of preclinical findings using cfDNA into a robust clinical test will require further technical considerations. Discovery of low-abundance somatic mutations among normal cfDNA poses challenges for rigorous clinical application. *AR* mutations detected by deep sequencing of plasma-derived cfDNA have been reported at allelic fractions as low as 0.11% [14]. Analytical optimization will be necessary to ensure specificity as deep sequencing has the potential for low level error that may be comparable to genuine low-abundant mutations. Cross platform analysis of low-abundant mutations using technologies such as ddPCR would offer insights to the validity of these low-abundant mutations, but secondary analysis is often limited due to sample depletion. Herein, we aimed to validate low-abundant *AR* mutations discovered by deep sequencing cfDNA from patients with mCRPC by ddPCR.

## RESULTS

### Patient cohort

We prospectively enrolled 11 patients treated at Johns Hopkins Hospital (Baltimore, MD) and Sibley Memorial Hospital (Washington D.C.) for mCRPC. Patients had histologically confirmed prostate adenocarcinoma, progressive disease despite androgen deprivation therapy, and documented metastatic disease by computed tomography (CT) or bone scan with technetium-99m-labeled methylene diphosphonate. Plasma-derived cfDNA was isolated from all patients prior to initiation of enzalutamide therapy. Patient characteristics at plasma collection are summarized in Table 1. Most patients had multiple therapies prior to collection and bone disease at collection. Patient response to enzalutamide is summarized in Supplementary Figure 2A-2D.

**Table 1:** Patient Characteristics

Characteristics	(n=11)
Age, years	
Median (range)	71 (41-90)
Race	
White	11
Black	0
Prior Treatment for Prostate Cancer, n (%)	
Radical Prostatectomy	2 (18)
Primary Radiation	2 (18)
ADT	7 (64)
Gleason Sum, n (%)	
≤7	0 (0)
≥8	9 (81.8)
Not Available	2 (18.2)
Prior Treatment for Metastatic Prostate Cancer, n (%)	
ADT Alone	4 (36.4)
ADT and Abiraterone	1 (9.1)
ADT, Abiraterone, and Chemotherapy	1 (9.1)
ADT and Chemotherapy	2 (18.2)
ADT, Chemotherapy, and Radiation	3 (27.3)
PSA, ng/ml	
Median (range)	46.6 (0.9-183.7)
Site of Metastases, n (%)	
Bone Metastases	9 (81.8)
Visceral Metastases	1 (9.1)
Lymph Lode Only	1 (9.1)
PSA Progression Free Survival, days	
Median (range)	168 (35-466)

### Deep NGS of *AR* from plasma-derived cfDNA

Plasma derived cfDNA from patients with mCRPC was amplified using the Qiagen Prostate targeted panel with Qiagen HotStarTaq and Qiagen HiFi Taq and then deep sequenced for *AR* mutations on the Illumina HiSeq. Plasma derived cfDNA from two healthy donors (one male and one female) was also amplified and sequenced. The *AR* (TGg>TGt/c) W742C hotspot mutation was detected in one patient at an allelic frequency of 0.46% (Table 2). The *AR* (aCT>gCT) T878A hotspot mutation was detected in one patient at an allelic frequency of 0.42% (Table 2). These mutations were not detected in cfDNA from the healthy controls (Table 2). Unexpectedly, the *AR* (tTC>cTC) F877L hotspot mutation was detected in approximately 45% of the patients (n=5/11) at allelic fractions between 0.20-0.28% (Table 2). While these allelic fractions were within previously reported ranges for *AR* hotspot mutations identified using similar technologies in comparable patients, the frequency of the *AR* F877L mutation in this cohort was over ten-fold higher than in previously published studies [14, 18, 19]. In addition, this mutation was also detected at a lower level in the healthy male control (Table 2). Other *AR* hotspot mutations, *AR* (CtC>CaC) L702H and *AR* (cAT>tAT) H875Y were not detected in any of the patient samples or controls (Table 2).

**Table 2:** NGS of *AR* using Qiagen Library Amplification Phred Quality Score ≥ 25

Sample ID	*AR* L702H (CtC>CaC)			*AR* W742C (TGg>TGt/c)			*AR* H875Y (cAT>tAT)			*AR* F877L (tTC>cTC)			*AR* T878A (aCT>gCT)		
	Mutant Read Count	Wild-type Read Count	Percent Mutant	Mutant Read Count	Wild-type Read Count	Percent Mutant	Mutant Read Count	Wild-type Read Count	Percent Mutant	Mutant Read Count	Wild-type Read Count	Percent Mutant	Mutant Read Count	Wild-type Read Count	Percent Mutant
JHU Pt 1	--	12,889	--	--	2,327	--	--	4,572	--	--	5,199	--	--	5,053	--
JHU Pt 2	--	13,987	--	--	3,853	--	--	6,061	--	--	7,384	--	--	7,158	--
JHU Pt 3	--	23,583	--	**21**	**4,519**	**0.46**	--	10,019	--	**26**	**11,152**	**0.23**	--	10,828	--
JHU Pt 4	--	16,374	--	--	3,867	--	--	6,983	--	--	8,215	--	**33**	**7,924**	**0.42**
JHU Pt 5	--	26,672	--	--	5,073	--	--	5,603	--	--	6,818	--	--	6,600	--
JHU Pt 6	--	440,962	--	--	108,069	--	--	236,296	--	--	283,867	--	--	274,418	--
JHU Pt 7	--	25,501	--	--	7,109	--	--	11,082	--	**36**	**13,386**	**0.27**	--	12,994	--
JHU Pt 8	--	21,1143	--	--	4,711	--	--	7,832	--	**25**	**8,863**	**0.28**	--	8,555	--
JHU Pt 9	--	17,129	--	--	4,529	--	--	7,317	--	--	9,055	--	--	8,792	--
JHU Pt 10	--	51,691	--	--	11,388	--	--	17,495	--	**51**	**20,322**	**0.25**	--	19,606	--
JHU Pt 11	--	19,265	--	--	3,140	--	--	6,621	--	**15**	**7,334**	**0.20**	--	7,134	--
Male Control	--	21,891	--	--	6,529	--	--	16,455	--	**30**	**17,609**	**0.17**	--	18,560	--
Female Control	--	37,134	--	--	9,913	--	--	33,242	--	--	35,441	--	--	35,279	--

### Validation of *AR* mutations from cfDNA by ddPCR

The unprecedented frequency of the *AR* F877L mutation in this cohort as well as its overall low allelic fraction and its occurrence in a healthy male control collectively indicate these mutations to be false positives. As the NGS workflow utilized in this study was designed to limit DNA contamination and the NGS data did not show evidence of sample to sample contamination, the *AR* F877L mutations were most likely not from contaminating DNA. Error can occur due to *Taq* polymerase infidelity during targeted library amplification prior to sequencing or during NGS and subsequent variant calling. As *AR* F877L mutations have been reported at low allelic frequencies and *AR* F877L was not detected in all patient samples or controls, we sought to query these samples by an alternate platform to determine if any were genuine mutations that were masked by low level sequencing error. We additionally sought to use an alternate platform to determine if this site is commonly prone to amplification mediated error. To do this, we examined samples by ddPCR with prior preamplification using high fidelity polymerases, NEB Phusion® or Invitrogen™ Platinum™ SuperFi™ (Table 3). Patient cfDNA, wild-type control genomic DNA, and a no template control were preamplified using Phusion® prior to ddPCR. Due to limited sample amount, the five positive samples by NGS for the *AR* F877L mutation, the one positive sample by NGS for the *AR* T878A mutation, wild-type genomic DNA, and a no template control were amplified by Platinum SuperFi™. Preamplified samples and controls and non-amplified controls were then examined for wild-type *AR* and the *AR* F877L hotspot mutation by ddPCR. Ten of the eleven samples preamplified by Phusion® were positive for the *AR* F877L (tTC>cTC) mutation by ddPCR at very low allelic fractions ranging from 0.007 to 0.033% (Table 4). Notably, Phusion® preamplified wild-type genomic DNA was also positive for the *AR* F877L (tTC>cTC) mutation by ddPCR while non-amplified wild-type genomic DNA was negative suggesting that the *AR* F877L mutation was introduced prior to ddPCR during preamplification with Phusion® (Table 4). DNA contamination is not likely the source of the *AR* F877L mutation as both the preamplified and the non-amplified no template controls were negative. In contrast to Phusion® preamplification, none of the Platinum SuperFi™ preamplified patient samples or wild-type control genomic DNA were positive for the *AR* F877L mutation (Table 4). Control genome equivalents assayed were comparable between the two polymerases suggesting that fidelity differences were not due to under representation or sensitivity. These data suggest that this specific *AR* locus is prone to PCR based error and that under these conditions at this locus, Platinum SuperFi™ has greater fidelity than Phusion®.

**Table 3:** Company Reported Polymerase Fidelity

	Qiagen HotStarTaq2 × 10^-5^	NEB Phusion^®^ High-Fidelity DNA PolymerasePhusion HF buffer	Invitrogen™ Platinum™ SuperFi™ DNA Polymerase
Components	Modified recombinant 94 kDa Taq DNA polymerase originally isolated from *Thermus aquaticus*	*Pyrococcus*-like proofreading enzyme fused with a processivity-enhancing domain.	Chemically engineered *Pyrococcus*-like enzyme
Company Reported Fidelity	Error Rate of 2 × 10^-5^	Error Rate of 4.4 × 10^-7^In Phusion HF buffer as reported by Finnzymes/Thermo Scientific	Greater than 100X Taq fidelityFidelity greater than Phusion®

**Table 4:** Comparison of NEB Phusion^®^ and Invitrogen™ Platinum™ SuperFi™ Preamplification of *AR* by ddPCR for *AR* F877L

Sample ID	*AR* F877L (tTC>cTC)ddPCRNEB Phusion^®^ reamplification			*AR* F877L (tTC>cTC)ddPCRInvitrogen™ Platinum™ SuperFi™ Preamplification		
	Mutant Droplet Count	Wild-type Droplet Count	Percent Mutant	Mutant Droplet Count	Wild-type Droplet Count	Percent Mutant
JHU Pt 1	5	18298	0.027	--	--	--
JHU Pt 2	8	31108	0.026	--	--	--
JHU Pt 3	5	27672	0.018	0	14824	0.000
JHU Pt 4	5	35181	0.014	0	14164	0.000
JHU Pt 5	11	32975	0.033	--	--	--
JHU Pt 6	0	41833	0.000	--	--	--
JHU Pt 7	7	68073	0.010	0	40588	0.000
JHU Pt 8	10	36587	0.027	0	10163	0.000
JHU Pt 9	6	25642	0.023	--	--	--
JHU Pt 10	4	59673	0.007	0	16317	0.000
JHU Pt 11	8	37478	0.021	0	17926	0.000
PreamplifiedWild-type Genomic DNA	11	30348	0.036	0	44104	0.000
Preamplified No Template Control	0	0	0.000	0	0	0.000
Non-AmplifiedWild-type Genomic DNA	0	36018	0.000	0	7761	0.000
Non-Amplified*AR* F877L Mutant Control DNA	964	0	100	810	0	100
Non-Amplified No Template Control	0	0	0.000	0	0	0.000

As Phusion® has been used for preamplification of cfDNA prior to ddPCR without reported false positives [13], we sought to determine if the *AR* locus encompassing the F877 codon was particularly susceptible to error during amplification or if preamplification with Phusion® broadly introduced a low level of error under these conditions. To do this, we examined both the adjacent codon on exon 8 for the *AR* T878A hotspot mutation and an upstream codon on exon 5 for the hotspot mutation *AR* W742C by ddPCR of Phusion® and Platinum SuperFi™ preamplified wild-type genomic DNA and patient cfDNA. Due to limited sample amount, we selected seven of the eleven patient samples including the five that were positive for the *AR* F877L mutation and the one that was positive for the *AR* T878A mutation by deep sequencing and wild-type genomic DNA for preamplification by Phusion® and Platinum SuperFi™ prior to ddPCR for wild-type *AR* and the *AR* T878A mutation. Control cfDNA from a patient with an *AR* T878A mutation was included to further validate the efficacy of the *AR* T878A ddPCR probe. Five of the seven Phusion® preamplified patient samples as well as the wild-type genomic DNA were also positive for the *AR* T878A mutation, but at significantly (*P*=0.02) lower allelic frequencies (0.004 to 0.011%) than the *AR* F877L mutation (Table 5). Decreasing preamplification cycle number from 22 to 12 did not eliminate the introduction of either the *AR* F877L or *AR* T878A mutations when wild-type genomic DNA was preamplified by Phusion® (Table 6). In contrast to Phusion® preamplification, all Platinum SuperFi™ preamplified JHU patient samples and wild-type genomic DNA were negative for the *AR* T878A mutation (Table 5). Interestingly, Patient 4, who was positive for the *AR* T878A mutation by NGS, was negative by ddPCR while the *AR* T878A positive control cfDNA was positive for the *AR* T878A mutation. Collectively, this suggests that the *AR* T878A mutation detected by NGS in JHU patient 4 was a false positive. Again, genome equivalents were comparable between assays suggesting that differences were not due to under representation or sensitivity.

**Table 5:** Comparison of NEB Phusion^®^ and Invitrogen™ Platinum™ SuperFi™ Preamplification of *AR* by ddPCR for *AR* T878A

Sample ID	*AR* T878A (aCT>gCT)ddPCRNEB Phusion^®^ Preamplification			*AR* T878A (aCT>gCT)ddPCRInvitrogen™ Platinum™ SuperFi™ Preamplification		
	Mutant Droplet Count	Wild-type Droplet Count	Percent Mutant	Mutant Droplet Count	Wild-type Droplet Count	Percent Mutant
JHU Pt 1	1	11707	0.009	0	41931	0.000
JHU Pt 3	1	15126	0.007	0	26745	0.000
JHU Pt 4	3	26983	0.011	0	30737	0.000
JHU Pt 7	0	12156	0.000	0	35931	0.000
JHU Pt 8	1	24205	0.004	0	27729	0.000
JHU Pt 10	0	51299	0.000	0	20279	0.000
JHU Pt 11	1	27082	0.004	0	32648	0.000
PreamplifiedWild-type Genomic DNA	2	36329	0.006	0	21511	0.000
PreamplifiedNo Template Control	0	0	0.000	0	0	0.000
Preamplified cfDNA from a patient with an *AR* T878A mutation detected by NGS	---	---	---	233	11890	1.960
Non-AmplifiedWild-type Genomic DNA	0	16132	0.000	0	24915	0.000
Non-Amplified*AR* T878A Mutant Control DNA	122	0	100	7502	0	100
Non-AmplifiedNo Template Control	0	0	0.000	0	0	0.000

**Table 6:** Comparison of NEB Phusion^®^ and Invitrogen™ Platinum™ SuperFi™ Preamplification of *AR* by ddPCR for *AR* Hotspot Mutations

Wild-typeGenomic DNA	NEB Phusion^®^PreamplificationPhusion^®^ HF Buffer(22 cycles)			NEB Phusion^®^PreamplificationPhusion^®^ HF Buffer(12 cycles)			Invitrogen™ Platinum™ SuperFi™Preamplification(22 cycles)		
	Mutant Read Count	Wild-type Read Count	Percent Mutant	Mutant Read Count	Wild-type Read Count	Percent Mutant	Mutant Read Count	Wild-type Read Count	Percent Mutant
*AR*F877LtTC>cTC	11	30348	0.036	1	8539	0.012	0	44104	0.000
*AR*T878AaCT>gCT	2	36329	0.006	1	9617	0.010	0	21511	0.000

We next examined patient samples and wild-type genomic DNA preamplified by Phusion® and Platinum SuperFi™ for wild-type *AR* and the *AR* hotspot mutation W742C. Notably, Patient 7, who was negative for *AR* W742C by deep sequencing, and wild-type genomic DNA were both negative by ddPCR when preamplified by either Phusion® or Platinum SuperFi™ (Table 7). Patient 3, who was positive for the *AR* hotspot mutation W742C by deep sequencing, was also positive by ddPCR using either Phusion® or Platinum SuperFi™ preamplified cfDNA (Table 7). Collectively, this suggests that *AR* (tTC>cTC) F877L and to a lesser extent *AR* (aCT>gCT) T878A are prone to error during Phusion® mediated PCR amplification while other *AR* loci such as *AR* (TGf>TGt/c) W742C may not be.

**Table 7:** Comparison of NEB Phusion^®^ and Invitrogen™ Platinum™ SuperFi™ Preamplification of *AR* by ddPCR for *AR* W742C

Sample ID	*AR*W742C (TGg>TGt/c)ddPCRNEB Phusion^®^ Preamplification			*AR*W742C (TGg>TGt/c)ddPCRInvitrogen™ Platinum™ SuperFi™ Preamplification		
	Mutant Droplet Count	Wild-type Droplet Count	Percent Mutant	Mutant Droplet Count	Wild-type Droplet Count	Percent Mutant
JHU Pt 3	4	25067	0.016	38	33611	0.113
JHU Pt 7	0	19753	0.000	0	15958	0.000
PreamplifiedWild-type Genomic DNA	0	17573	0.000	0	37069	0.000
PreamplifiedNo Template Control	0	0	0.000	0	0	0.000
Non-AmplifiedWild-type Genomic DNA	0	31225	0.000	0	31225	0.000
Non-Amplified*AR* W742C Mutant Control DNA	10602	0	100	10602	0	100
Non-AmplifiedNo Template Control	0	0	0.000	0	0	0.000

Since prior pre-clinical and clinical studies [14, 19–22] support that *AR* F877L and *AR* T878A may mediate resistance to androgen-AR axis therapies such as enzalutamide, we examined for an association of *AR* gene aberrations including *AR* LBD hotspot mutations and *AR* amplification with enzalutamide response. *AR* amplification was determined by ddPCR (Supplementary Figure 1). Analyses of validated *AR* gene aberrations (*AR* hot spot mutations and *AR* amplification) support that *AR* gene aberrations trend, but not significantly, with a worse PSA response as measured as best PSA percent change (>0 versus ≤0) by Fisher's exact test (Supplementary Figure 2A). Significance was further reduced by inclusion of the false positive *AR* F877L and *AR* T878A mutations detected by NGS (Supplementary Figure 2C). Kaplan-meier PSA progression-fee survival curves support that *AR* gene aberrations were significantly associated with a shorter time to PSA progression on enzalutamide by Log-rank (Mantel-Cox) tests (Supplementary Figure 2B). Significance was slightly decreased by inclusion of false positive *AR* F877L and *AR* T878A mutations detected by NGS (Supplementary Figure 2D). While the small cohort size limits robust conclusions pertaining to the association of *AR* gene aberrations with PSA response or PSA progression free survival, these findings are consistent with prior findings [14, 15, 17].

## DISCUSSION

Mainstay treatment strategies for newly diagnosed metastatic prostate cancer exploit prostate cancer addiction to AR signaling by inhibiting the androgen/AR axis with androgen deprivation therapies (ADT). While ADT is initially effective in most men, prostate cancers almost universally recur after a variable amount of time leading to mCRPC. Despite androgen blockade, the majority of newly diagnosed mCRPC are thought to have continued dependence on androgen signaling. Similar to first line ADT, next generation therapies for mCRPC such as abiraterone and enzalutamide also function by inhibiting androgen/AR signaling. Somatic alterations in *AR* including splice variants [23–26] and genetic alterations such as amplification and LBD mutations [14, 15, 17] have been associated with resistance to these therapies. Somatic point mutations in *AR* have been shown to occur in 5-18% of patients with mCRPC [12, 27, 28] and account for nearly 60% of cases if combined with *AR* amplification [12]. Consequently, there is much enthusiasm in validating *AR* status as a biomarker to guide therapeutic decisions for men with mCRPC.

Evaluating and monitoring *AR* status in patients with mCRPC using traditional metastatic biopsies poses several clinical challenges including cost, patient discomfort, sample collection, and lack of ease for serial analyses. Single site biopsies may also not account for tumor heterogeneity. Due to these limitations, many rapidly developing technologies have focused on revolutionizing the detection of tumor specific genetic alterations in the blood as a “liquid biopsy”. Use of cfDNA as a tumor analyte combined with NGS as a platform for detection show great promise in the clinic as a means for mutation detection and monitoring. While these technologies have demonstrated proof of principle, further optimization and validation is necessary to define parameters for sensitivity and specificity. Concerns currently exist pertaining to the rate of false positives and the associated potential clinical ramifications. NGS of cfDNA for the detection of tumor specific mutations often involves polymerase based amplification which can introduce errors. While NGS approaches such as SAFESeqS [29] and Duplex sequencing [30] may dramatically limit sequencing based false positives, these technologies have yet to be widely implemented. Thus, rigorous optimization and standardization of these technologies will be needed prior to use for clinical decision making.

**Figure 1:**
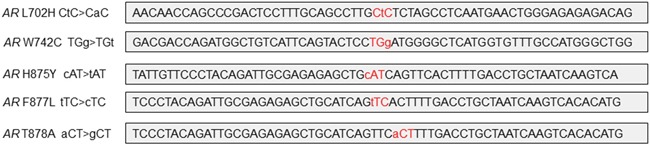
Genomic region surrounding loci of *AR* hotspot mutations

To begin to address issues pertaining to false positive rate and lower bound threshold for detection, we sought to identify mutations in *AR* by NGS of plasma-derived cfDNA from patients with mCRPC and then to cross-platform validate by ddPCR. Our findings suggest variability in polymerase fidelity at specific *AR* LBD hotspot loci. We demonstrate that some high fidelity polymerases used for preamplification prior to NGS and ddPCR can introduce low level mutations at *AR* LBD hotspot F877L and to a lesser extent at *AR* T878A. Interestingly, other *AR* LBD hotspots such as *AR* W742C did not show false positives by any of the tested polymerases. Sequence differences between these loci were not readily appreciable as they contain comparable GC content and single and dinucleotide repeats (Figure 1). The experimental error rates at the adjacent LBD hotspot loci, 877 and 878, far exceeded the reported overall error rate for these polymerases, thereby suggesting that *AR* F877L and *AR* T878A may be particularly prone to amplification error. These data support that previously unappreciable areas of the genome may be more susceptible to NGS preamplification based errors.

Clinical data and laboratory based studies support that both the *AR* T878A and the *AR* F877L mutations have potential clinical significance. *AR* T878A was originally identified in the LNCaP prostate cancer cell line [31] while *AR* F877L was identified using an *in vitro* screen for resistance mechanisms to anti-androgens [21]. *AR* F877L and *AR* T878A have been detected in patients with mCRPC [14, 18, 19, 22]; however, the *AR* F877L mutation has only been detected by NGS of cfDNA from mCRPC patients [14, 18, 19] and has yet to be reported in a metastatic lesion by NGS [12, 27, 28]. Laboratory based studies support that both AR T878A [19] and AR F877L [18, 19, 22] may confer resistance to enzalutamide and ARN-509. Thus reliable detection of these and other *AR* LBD mutations may impact decisions in the clinic.

This study highlights the need for rigorous protocol optimization for the detection of mutations in cfDNA by NGS. We demonstrate that some loci may be more susceptible to polymerase based errors and thereby stress the need for loci specific polymerase optimization and standardization, use of proper controls, variant calling, and validation. While use of more stringent variant calling criteria would exclude the F877L false positives detected by NGS and improve specificity, it would consequently decrease sensitivity by also excluding genuine low abundant mutations such as that found in Patient 3. Currently, an accepted standard or platform for using cfDNA for mutation detection does not exist. These studies underscore the need to establish and to validate protocols for mutation detection and monitoring in cfDNA by NGS. By doing so, integration of these technologies will ultimately advance patient care.

## MATERIALS AND METHODS

### Patients and sample collection

Human subject research was approved by the Johns Hopkins Medicine Institutional Review Board (IRB). We prospectively enrolled patients diagnosed with metastatic castration resistant prostate cancer (mCRPC) (n=11) between September 2014 and April 2015 prior to initiation of enzalutamide. PSA progression was determined at the date of the first PSA rise that was followed by a subsequent PSA rise or at the date of physician determined change of therapy. All patients provided written and informed consent to participate in the study. Two healthy controls also participated in the study. Three 10 ml blood samples were collected in Streck BCT tubes prior to therapy initiation, stored at room temperature, and processed for plasma isolation within 24 hours. To optimize patient sample integrity and to limit DNA contamination, plasma was extracted in a bleach and UV cleaned hood specifically for plasma extraction in a room dedicated for blood processing, storage, and cell-free DNA isolation. Plasma was extracted from blood by centrifugation for 10 minutes at 1500 x g followed by a second centrifugation for 10 minutes at 3000 x g as previously described [13, 32, 33]. Plasma was stored at -80°C in 1mL aliquots. In a dedicated bleach and UV cleaned hood, cell-free DNA was extracted from plasma using the QIAamp Circulating Nucleic Acid Kit (Qiagen) per the manufacturer's protocol. To limit contamination, only one patient sample was processed at a time. Cell-free DNA was quantified using the Quant-iT PicoGreen dsDNA assay kit (Invitrogen) as per the manufacturer's protocol.

### Deep next generation sequencing

GeneRead™ DNAseq Targeted Panel V2 (Qiagen) was used to prepare libraries from cfDNA for NGS as per the manufacturer's protocol. NGS libraries were prepared in dedicated bleach and UV cleaned hood. Samples were PCR amplified using Qiagen HotStar Taq as per the manufacturer's protocol. Libraries were amplified using Qiagen HiFi PCR Master Mix for 4 cycles. NGS was performed on the Illumina Hi-Seq with an average depth of coverage of 10,000x. Data was aligned to hg19 using bwa-0.7.7 (mem function). SAMtools was used to filter reads by quality (Phred ≥ 25 and mapQ >18 were included). The threshold for non-reference reads was 15 and single strand variant calls, non-reference reads less than or equal to 0.15%, non-reference reads less than twice the next highest non-reference frequency, variants in more than 50% of the samples, and variants over 50% of the allelic fraction were excluded. *AR* hotspot variants were additionally examined using the Integrative Genomics Viewer (IGV) http://www.broadinstitute.org/igv/. Variants that passed these filters were then validated by ddPCR. Variant calls were made independent/blinded to outcome data.

### Droplet digital PCR

Control genomic DNA (Promega) was digested with MseI (NEB). Genomic DNA, patient cfDNA, and water (no template control) were PCR amplified using Phusion® with Phusion® HF buffer (NEB) or Platinum SuperFi™ (Invitrogen) at loci surrounding *AR* amino acid 742 and *AR* amino acid 877/878 for either 12 or 22 cycles. PCR amplification primer sequences are located in Supplementary Table 1. Three independent preamplification PCR reactions of 1 ng DNA each were pooled for each sample and control. Similarly, three independent preamplification PCR reactions were pooled for each no template control. Preamplified samples and controls were purified prior to ddPCR using the MinElute PCR Purification kit (Qiagen) and eluted in RNAase free water according to the manufacturer's instructions. Non-amplified *AR* W742C, *AR* F877L, and *AR* T878A mutant control DNA for ddPCR were double-stranded purified DNA gBlocks®Gene Fragments (IDT) of approximately 400 base pairs that were reconstituted in RNAase free water. The non-amplified no template control had RNAase free water without template.

Dual labeled (FAM or HEX) fluorescent-quencher hydrolysis probes (IDT) were designed for *AR* hotspot mutations (W742C, F877L, and T878A) and their respective wild-type loci. Sequences for ddPCR primers and probes are located in Supplementary Table 1. Wild-type control genomic female DNA (Promega) digested with MseI and mutant DNA gBlocks®Gene Fragments (IDT) were used to optimize primer/probe conditions. Probes to *AR* (ABI Hs04121925_cn FAM), *ZXDB* (ABI Hs02220689_cn FAM), and *NSUN3* (Bio-Rad dHsaCP2506682) were used to determine *AR* copy number. Samples with *AR* copy number greater than or equal to 1.9 were defined as having increased *AR* copy number. Wild-type control genomic female and male DNA (Promega) digested with MseI for *AR* or HaeIII for *ZXDB* were used for *AR* copy number controls. Cell-free DNA from healthy male and female donors was also used as a control. Droplet digital PCR (Bio-Rad) was performed in a dedicated UV equipped hood and according to the manufacturer's protocol. Total WT and mutant DNA molecules were quantified by the QX200 Droplet Reader software. Results for each mutation analysis were recorded as the summation of four or more replicates.

### Statistical analysis

Statistical Analyses were performed using GraphPad Prism software. All statistical tests were two-sided and *P* values less than 0.05 were considered statistically significant.

## SUPPLEMENTARY MATERIALS FIGURES AND TABLES


