# Supplementary material for: Detection fidelity of AR mutations in plasma derived cell-free DNA
Source: Oncotarget. 2017 Jan 31;8(9):15651–62. doi: 10.18632/oncotarget.14926 (PMC5362513; doi:10.18632/oncotarget.14926)
Supplement: Supplementary file 1 [file oncotarget-08-15651-s001.pdf]

# Detection fidelity of *AR* mutations in plasma derived cell-free DNA

## SUPPLEMENTARY FIGURES AND TABLES

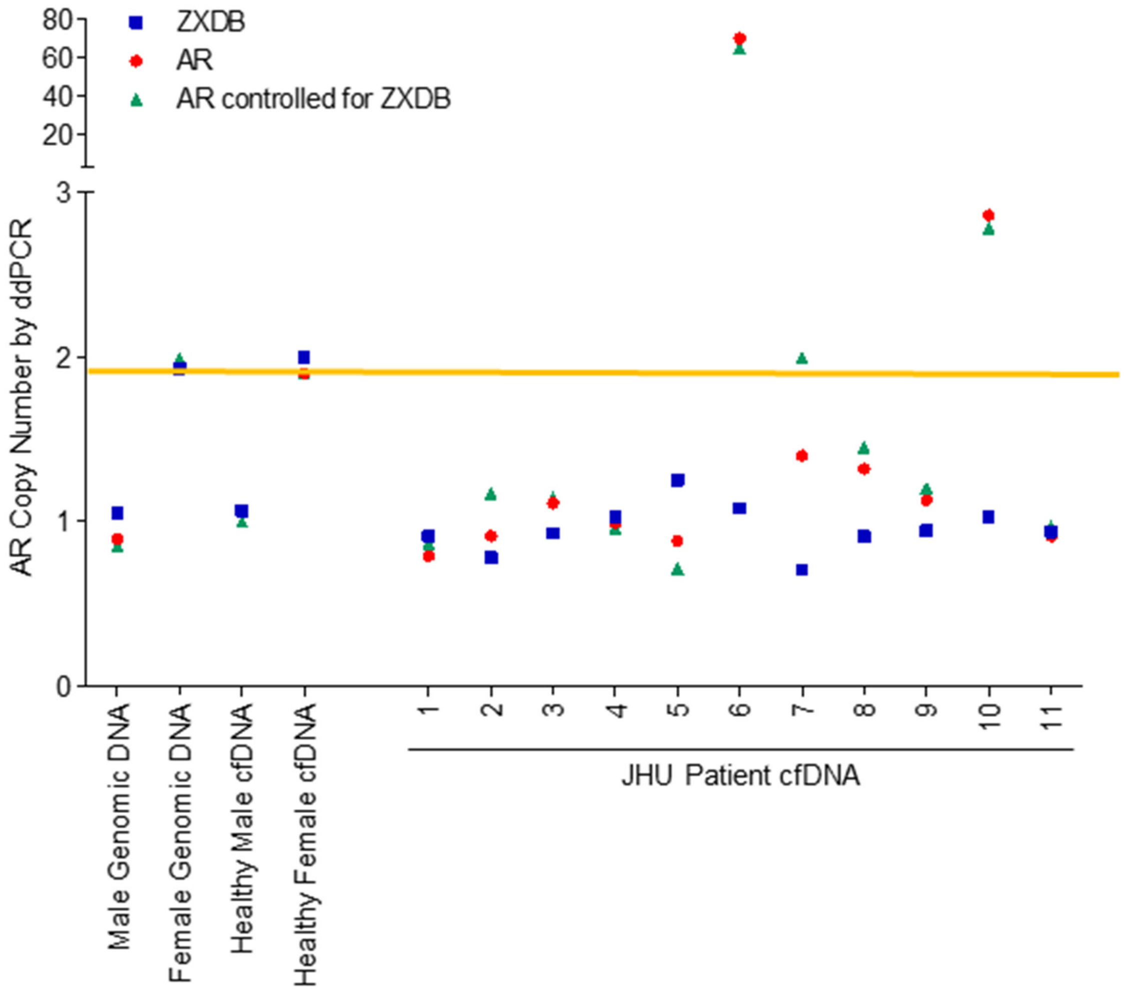


**Supplementary Figure 1: *AR* copy number detected in pre-therapy cfDNA as determined by ddPCR.** Genomic DNA and cfDNA from males and females was used as a control.

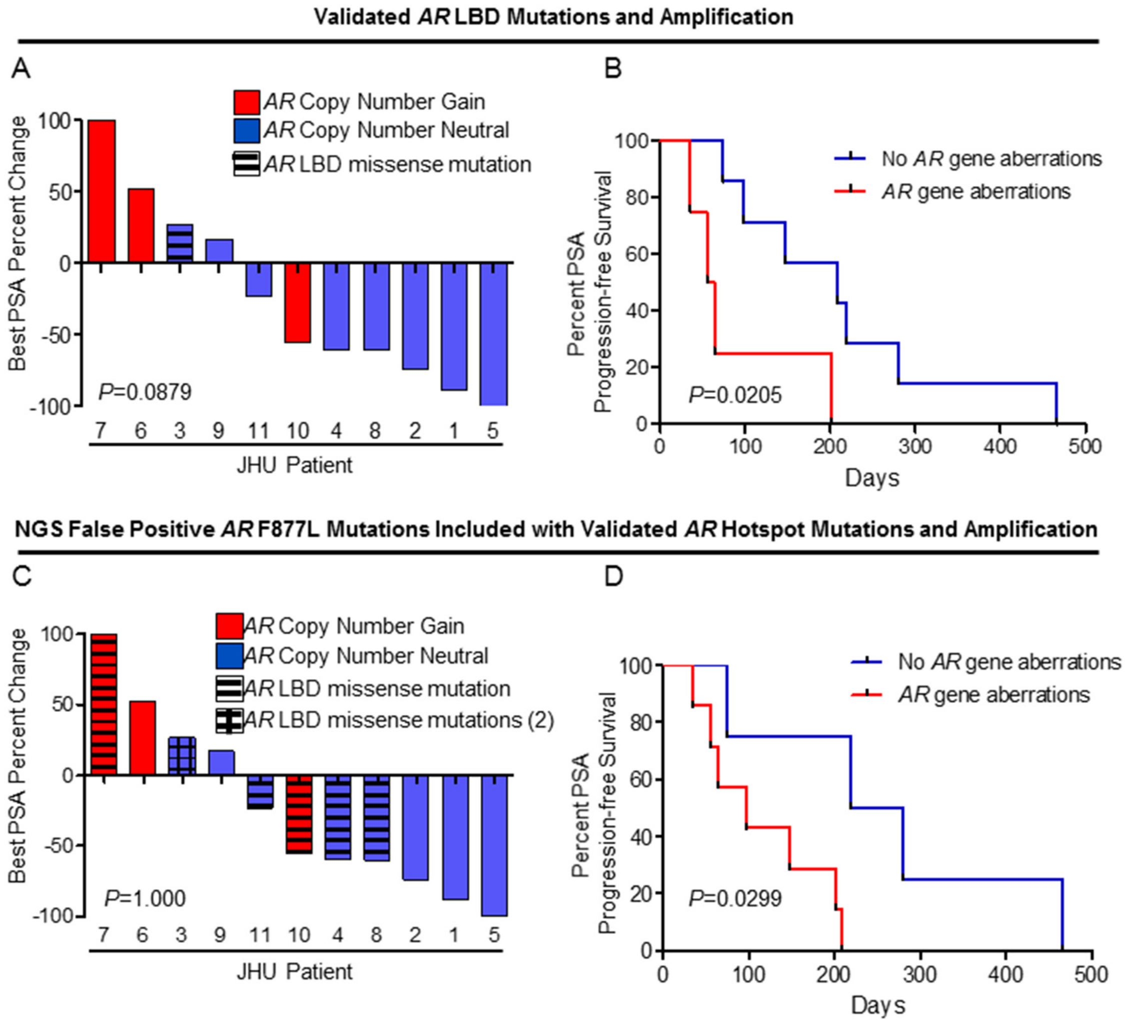


**Supplementary Figure 2: *AR* gene aberrations detected in pre-therapy cfDNA and response to enzalutamide. A.** Waterfall plot of best PSA response according to validated *AR* gene aberration status in pre-therapy cfDNA. *AR* LBD mutations detected by NGS in pre-therapy patient cfDNA and validated by ddPCR. *AR* copy number in pre-therapy patient cfDNA as determined by ddPCR. Significance determined by Fisher's exact test. **B.** Kaplan-Meier analysis of PSA progression-free survival according to validated *AR* gene aberration status in pre-therapy cfDNA. Significance determined by log-rank test. **C.** Waterfall plot of best PSA response according to false-positive and validated *AR* gene aberration status in pre-therapy cfDNA. *AR* LBD mutations detected by NGS in pre-therapy patient cfDNA and validated by ddPCR. *AR* copy number in pre-therapy patient cfDNA as determined by ddPCR. Significance determined by Fisher's exact test. **D.** Kaplan-Meier analysis of PSA progression-free survival according to false-positive and validated *AR* gene aberration status in pre-therapy cfDNA. Significance determined by log-rank test.

**Supplementary Table 1: Sequences of PCR Preamplification Primers, ddPCR Primers, and ddPCR Probes**

| **PCR Preamplification Primers Sequences** | **Forward** | **Reverse** |
|---|---|---|
| *AR* 742 | TAGCTCAACCCGTCAGTA | GCATCCTGGAGTTGACATT |
| *AR* 877/878 | TGATCTCTGCCATCATTTCC | CTACAGATTGCGAGAGAGC |
| **ddPCR Primers Sequences** | **Forward** | **Reverse** |
| *AR* 742 | TTCTTCTCCAGGCTTCCG | ATTGGTGAAGGATCGCCA |
| *AR* 877/878 | CTACAGATTGCGAGAGAG | TGATCTCTGCCATCATTTCC |
| **ddPCR Probes Sequences** | **WT probe** | **MUT probe** |
| *AR* W742C | 5HEX\TGTCATTCA\ZEN\ GTACTCCTGGATGG\3IABkFQ | 56-FAM\TGTCATTCA\ZEN\ GTACTCCTGCATGG\3IABkFQ |
| *AR* F877L | 5HEX\ATCAGTTCA\ZEN\ CTTTTGACCTGCTA\3IABkFQ | 56-FAM\ATCAGCTCA\ZEN\ CTTTTGACCTGCTA\3IABkFQ |
| *AR* T878A | 5HEX\ATCAGTTCA\ZEN\ CTTTTGACCTGCTA\3IABkFQ | 56-FAM\ATCAGTTCG\ZEN\ CTTTTGACCTGCTA\3IABkFQ |

All primers are read 5’ to 3’
